# Reversal of the glycolytic phenotype of primary effusion lymphoma cells by combined targeting of cellular metabolism and PI3K/Akt/ mTOR signaling

**DOI:** 10.18632/oncotarget.6315

**Published:** 2015-11-06

**Authors:** Laura Mediani, Federica Gibellini, Jessika Bertacchini, Chiara Frasson, Raffaella Bosco, Benedetta Accordi, Giuseppe Basso, Massimo Bonora, Maria Luisa Calabrò, Adriana Mattiolo, Gianluca Sgarbi, Alessandra Baracca, Paolo Pinton, Giovanni Riva, Enrico Rampazzo, Luca Petrizza, Luca Prodi, Daniela Milani, Mario Luppi, Leonardo Potenza, Anto De Pol, Lucio Cocco, Silvano Capitani, Sandra Marmiroli

**Affiliations:** ^1^ Department of Surgery, Medicine, Dentistry and Morphology, University of Modena and Reggio Emilia, Modena, Italy; ^2^ Department of Woman's and Child's Health and Institute of Pediatric Research - Città della Speranza Foundation, University of Padova, Padova, Italy; ^3^ Department of Morphology, Surgery and Experimental Medicine Section of Pathology, Oncology and Experimental Biology, University of Ferrara, Ferrara, Italy; ^4^ Immunology and Molecular Oncology, Veneto Institute of Oncology, IOV IRCCS, Padova, Italy; ^5^ Department of Biomedical and NeuroMotor Sciences, University of Bologna, Bologna, Italy; ^6^ Department of Medical and Surgical Sciences, Section of Hematology, University of Modena and Reggio Emilia, AOU Policlinico, Modena, Italy; ^7^ Department of Chemistry, University of Bologna, Bologna, Italy; ^8^ Department of Morphology, Surgery and Experimental Medicine, Section of Anatomy and Histology and LTTA Center, University of Ferrara, Ferrara, Italy

**Keywords:** PEL/non-Hodgkin lymphoma, glycolyis inhibitors, Warburg phenotype, hypoxia, PI3K/Akt/mTOR inhibitors

## Abstract

PEL is a B-cell non-Hodgkin lymphoma, occurring predominantly as a lymphomatous effusion in body cavities, characterized by aggressive clinical course, with no standard therapy. Based on previous reports that PEL cells display a Warburg phenotype, we hypothesized that the highly hypoxic environment in which they grow *in vivo* makes them more reliant on glycolysis, and more vulnerable to drugs targeting this pathway. We established here that indeed PEL cells in hypoxia are more sensitive to glycolysis inhibition. Furthermore, since PI3K/Akt/mTOR has been proposed as a drug target in PEL, we ascertained that pathway-specific inhibitors, namely the dual PI3K and mTOR inhibitor, PF-04691502, and the Akt inhibitor, Akti 1/2, display improved cytotoxicity to PEL cells in hypoxic conditions. Unexpectedly, we found that these drugs reduce lactate production/extracellular acidification rate, and, in combination with the glycolysis inhibitor 2-deoxyglucose (2-DG), they shift PEL cells metabolism from aerobic glycolysis towards oxidative respiration. Moreover, the associations possess strong synergistic cytotoxicity towards PEL cells, and thus may reduce adverse reaction *in vivo*, while displaying very low toxicity to normal lymphocytes. Finally, we showed that the association of 2-DG and PF-04691502 maintains its cytotoxic and proapoptotic effect also in PEL cells co-cultured with human primary mesothelial cells, a condition known to mimic the *in vivo* environment and to exert a protective and pro-survival action. All together, these results provide a compelling rationale for the clinical development of new therapies for the treatment of PEL, based on combined targeting of glycolytic metabolism and constitutively activated signaling pathways.

## INTRODUCTION

Primary effusion lymphoma (PEL) is a rare subtype of B-cell non-Hodgkin lymphoma (B-NHL) whose primary etiological agent is Kaposi's sarcoma-associated herpesvirus human herpesvirus 8 (KSHV, or HHV-8), and is often co-infected with Epstein-Barr virus (EBV) [1–5]. This malignancy is characterized by a very aggressive clinical course, with median survival times of a few months upon conventional chemotherapy. Viral gene products have been reported to dysregulate cellular signaling pathways and to favor survival. Viral proteins such as K1 and vGPCR can activate the phosphatidylinositol-3-kinase/Akt/mammalian target of rapamycin (PI3K/Akt/mTOR) pathway in B-lymphocytes and its constitutive activation was suggested to play a critical role in PEL cells growth and survival [6–8]. The efficacy of the mTOR inhibitor rapamycin, Sirolimus, in PEL cells in culture and in a xenograft PEL model, however, was limited by the rapid emergence of drug resistance [9]. Vertical targeting of the pathway at multiple levels by the dual PI3K/mTOR inhibitor NVP-BEZ235 gave more promising results [10]. In addition to its central role in promoting cell survival and inhibiting apoptosis [11–15], the alteration of intracellular signaling through the PI3K/Akt pathway is deemed crucial to achieve a cancer metabolic phenotype through the Warburg effect [16–20], i.e. the upregulation of glycolysis that allows the branching off of glycolytic intermediates to different anabolic pathways to sustain the higher demand of the transformed cells for metabolic inputs to promote proliferation [21–25]. Compared to primary B-cells, PEL cell lines are characterized by a glycolytic phenotype [26]; this in turn makes PEL cells sensitive to the glycolysis inhibitor 2-deoxyglucose (2-DG) under normoxic conditions [26, 27]. It should however be reminded that, *in vivo*, PEL develops in an hypoxic environment [28, 29], the body cavities. In our study we investigated the efficacy of a panel of glycolysis inhibitors against PEL cell lines in both normoxic and hypoxic conditions. On the other hand, because cellular responses to hypoxia may reduce chemotherapeutic sensitivity through diverse mechanisms acting on survival pathways, we addressed the effectiveness of drugs targeting various levels of the PI3K signaling cascade. Finally, we determined whether inhibition of both glycolysis and PI3K/Akt/mTOR-mediated survival results in a synergistic cooperation to induce more potent apoptosis and metabolic rerouting.

## RESULTS

### Glycolysis inhibitors are efficacious against PEL cell lines

**Figure 1:**
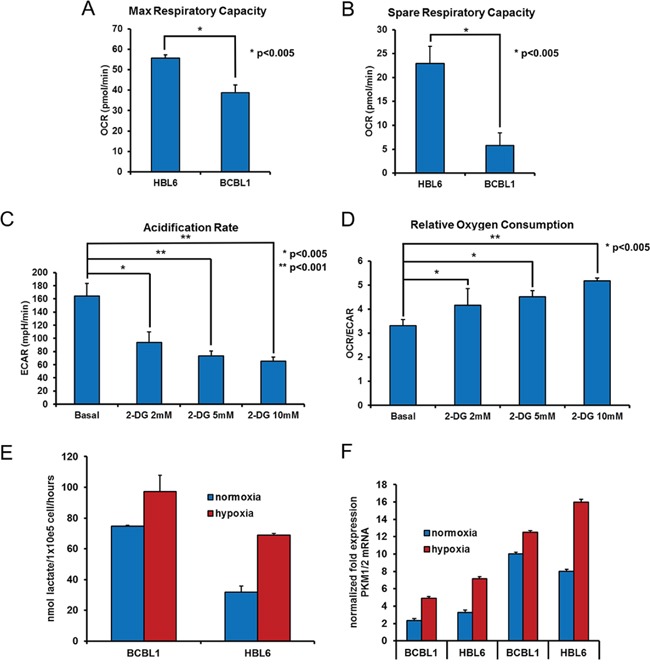
Hypoxia increases the glycolytic flux in PEL cells Before the assay, the cells were counted and seeded at 150.000 cell/well in XF96 culture plates. The oxygen consumption rate (OCR) was analyzed in real time by the XF96 analyzer in normoxia, under basal conditions and after sequential addition to the assay medium, through injection ports in the sensor cartridge, of 2 μM oligomycin A, 1 μM FCCP, and 1 μM rotenone + 1 μM antimycin A (final concentrations), to determine max respiratory capacity (OCR after injection of 1 μM FCCP) **A.** and spare respiratory capacity (OCR after injection of 1 μM FCCP minus basal OCR) **B.** Acidification of the growth medium (ECAR) of BCBL1 cells was measured in the absence (basal) or presence of 2-DG for 24 hours, as indicated. Addition of 2-DG to the medium drops ECAR by about 70%, due to HXK inhibition **C.** while the Relative Oxygen Consumption is shown by the OCR/ECAR ratio **D.** The level of lactate in the culture media of BCBL1 and HBL6 cells grown in normoxia or in hypoxia for 24 hours was measured as described in Methods. Proliferating cells grown in hypoxia divert glucose to lactate, resulting in increased lactate production. The data are expressed as the mean ± S.D. of three different replicates **E.** Cell pellets from the above experiment were analyzed by RT-PCR for the expression of the glycolysis rate-limiting enzymes PKM1 and PKM2 **F.** Where indicated, paired *t*-Test was performed on each mean value. p = 0.05.

PEL cells were claimed to display a Warburg phenotype, reflected in an elevated glycolytic activity under normoxic conditions, and inhibition of glycolysis was reported to be sufficient to reduce cell viability [26]. To define the metabolic profile of two representative PEL cell lines, BCBL1 and HBL6, we assessed real-time cellular respiration using the XF Extracellular Flux Analyser upon sequential injection of specific mitochondrial chemical probes to the culture medium [30]. First, the decrease in oxygen consumption rate (OCR) following addition of the complex V inhibitor, oligomycin, was used to calculate ATP-linked respiration and proton leak respiration. Next, carbonyl cyanide-p-trifluoromethoxyphenyl-hydrazon (FCCP), a protonophore, was added to collapse the inner membrane gradient, driving the electron proton chain to function to its maximal rate, and maximal respiratory capacity was calculated by subtracting non-mitochondrial respiration from the FCCP OCR. Finally, addition of the complex III inhibitor antimycin A, together with the complex I inhibitor rotenone, resulted in the arrest of the electron proton chain function, allowing quantification of non-mitochondrial respiration. The mitochondrial reserve capacity was then calculated by subtracting basal respiration from maximal respiratory capacity (Supplementary Figure S1A). The maximal respiratory capacity, elicited by exposure to FCCP, appeared much more pronounced in HBL6 compared to BCBL1 (Figure 1A). Besides, the spare respiratory capacity, an index of the efficiency of the electron transport chain to respond to energy demand and a predictor of the ability of cells to resist stressful conditions [31], reported to be reduced by oncogenic transformation [32, 33], was significantly lower in BCBL1 compared to HBL6 (Figure 1B). This reduced mitochondrial reserve capacity suggests that BCBL1 cells are more prone to increase glycolysis in hypoxic condition. Thus, we next estimated the extracellular acidification rate (ECAR), which correlates with the rate of glycolysis because of the by-product lactic acid, in BCBL1 cells. We observed that these cells indeed display a high basal aerobic glycolysis, assessed by ECAR (Figure 1C), together with low oxidative phosphorylation (Figure 1D). In the presence of the well-known hexokinase (HXK) inhibitor 2-DG [34–36], we observed reduction in extracellular acidification, due to the block of glycolytic flux, as expected (Figure 1C). Interestingly the ratio between OCR and ECAR in basal condition was significantly increased in presence of 2-DG compared to glucose only (Figure 1D). This value is an index of the relative contribution of glycolysis or mitochondrial activity on cellular metabolism and suggests that, in presence of 2-DG inhibition, a reversal of the Warburg effect is occurring [37]. Indeed as ECAR dropped by about 70%, the relative OCR level progressively increased, suggesting that mitochondria respiration is fueled through an alternative carbon source (Figure 1D). To substantiate this observation, we performed an indirect evaluation of the mitochondrial ATP production assessing the mitochondrial membrane potential fluctuations in response to oligomycin. In the presence of 2-DG, mitochondrial hyperpolarization boosted up, indicating an augmented production of mitochondrial ATP, which inversely correlates with the Warburg effect (Supplementary Figure S1B).

**Figure 2:**
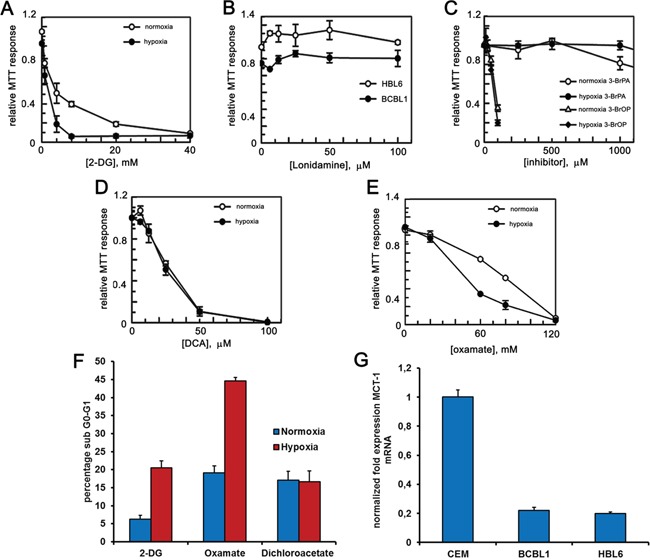
PEL cells are sensitive to glycolytic inhibitors BCBL1 and HBL6 cells were treated for 24 hours with increasing concentrations of 2-DG **A.** lonidamine **B.** 3-BrPA and 3-BrOP **C.** DCA **D.** oxamate **E.** MTT assays were performed in triplicates. The graphs show the mean ± S.D. **F.** Quantification of sub G0-G1 cells was carried by PI staining followed by flow cytometry analysis, upon treatment with 2-DG (2 mM), oxamate (40 mM) and DCA (30 μM) for 24 hours under hypoxia or normoxia. **G.** mRNA level of the glucose transporter MCT-1 was measured by RT-PCR in HBL6, BCBL1 and CEM cell lines.

**Figure 3:**
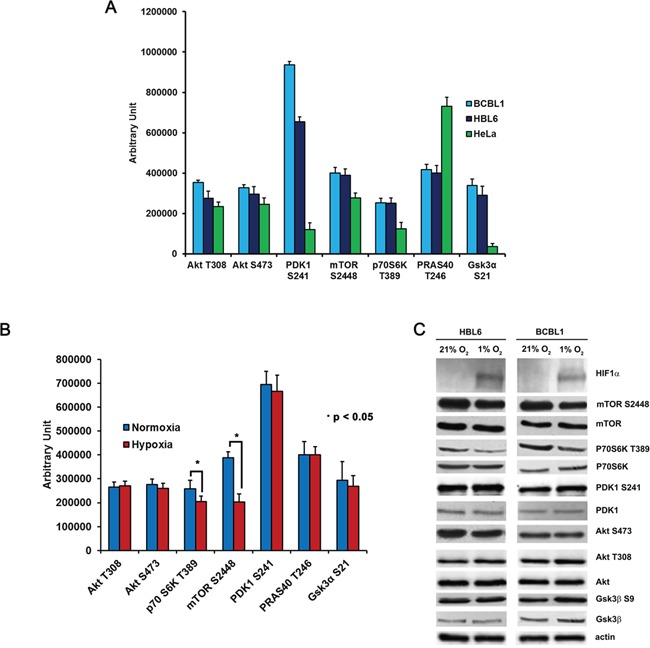
Hypoxia affects the PI3K/Akt/mTOR pathway at the mTORC1 level **A.** Activation of the PI3K/Akt/mTOR pathway was analyzed by RPPA in BCBL1 and HBL6 cells, and phosphorylation of key molecules was compared with HeLa cells. **B.** RPPA comparison of the phosphorylation status of the indicated signaling molecules in BCBL1 cells grown in normoxia or in hypoxia. **C.** Equal amounts of cellular protein extracts from (B) were resolved by SDS-PAGE and probed with the indicated antibodies. PDK1 S241, Akt T308, Akt S473 are a readout of Akt downstream signaling activation. P70S6K T389 is a readout of mTOR activation. HIF1α was is a readout of hypoxia. Equal loading was confirmed by anti-β-actin.

PEL cells grow in body cavities, i.e. in hypoxic conditions [28, 29]. It was therefore tempting to ask whether adaptation to such hostile setting makes PEL cells even more reliant on glycolysis for their energy requirements, and thus more vulnerable to inhibitors that target this metabolic pathway. Accordingly, we analyzed the glycolytic flux of PEL cells cultured in low oxygen. A boost in the secretion of lactate in the culturing medium (Figure 1E), paralleled by an increase in the mRNA expression of glycolytic enzymes pyruvate kinase PKM1 and PKM2 (Figure 1F), suggested that growing in low oxygen increases the glycolytic metabolism of these cells, although this might be due also to an hypoxia-dependent increase of the lactate transporter, the monocarboxylate transporter 1 MCT1 [38], whose expression in these cells is indeed low (Figure 2G). On these bases, we decided to analyze the cytotoxic effect of 2-DG on the above cell lines cultured in normoxia or hypoxia. Cells were incubated for 24 hours with increasing concentrations of 2-DG, followed by MTT assay. As expected, cell viability was inhibited in a dose-dependent manner by 2-DG (Figure 2A). Besides, hypoxia largely potentiated cell sensitivity to 2-DG [39], dropping cell viability at a lower dose (Figure 2A). Next, we compared the effect of 2-DG with that of molecules targeting different steps of glycolysis [40]. These included 3-bromo-pyruvate (3-BrPA) [41, 42], which targets glyceraldehyde-3-phosphate dehydrogenase (GAPDH) and HXK [43]; oxamate, which inhibits lactate dehydrogenase (LDH) [44, 45], and dichloroacetate (DCA), which acts instead by reversing the cancer-associated suppression of pyruvate dehydrogenase (PDH) through inhibition of pyruvate dehydrogenase kinase, thereby promoting the mitochondrial oxidation of pyruvate [46], and lonidamine [42, 47, 48], which inhibits HXK. To exclude potential alteration of cellular sensitivity to cytotoxic drugs due to a reduction in their ability to proliferate, viability of untreated cells was assessed by MTT after culturing for 24 hours in oxygen-deprived or normoxic conditions and found to be not statistically significant (average log2 fold change between the hypoxia and normoxia MTT values 0.074 ± 2.56; 2-tailed paired *t*-test of hypoxia versus normoxia values = 0.48). Lonidamine (Figure 2B) and 3-BrPA (Figure 2C) had no effect on cell viability even at very high concentration, of no clinical significance, and longer incubation times (not shown). Importantly, however, viability was markedly diminished in both cell lines following treatment with DCA (Figure 2D) and oxamate (Figure 2E). In the case of oxamate, the effect was further strengthened by hypoxia, similar to what observed with 2-DG (Table 1A). Thus, the ability of 2-DG, oxamate and DCA to cause apoptotic cell death was explored measuring the proportion of sub-G1 cells by flow cytometry. Cells cultured either in hypoxia or in normoxia had comparable basal level of apoptosis (about 5%). However, upon administration of equal doses of drugs, cells cultured in hypoxia in the presence of 2-DG or oxamate displayed more abundant apoptosis (Figure 2F), whereas, similar to what observed in the viability assay, DCA induced the same proportion of apoptosis in both normoxia and hypoxia (Figure 2F). Besides, we speculated that as both 3-BrPA and Lonidamine are transported into the cell through MCT1 [49, 50], these cell lines might express low amounts of the MCT1 protein and would be for this reason inherently resistant even to high concentration of the drugs. RT-PCR analysis confirmed that this is indeed the case, as both HBL6 and BCBL1 cells display very low levels of MCT1 transcript, compared to that found in cell lines sensitive to 3-BrPA such as the lymphoblastic leukemia cell line CEM (Figure 2G). Thus, because of its better cell permeability and chemical stability, the ester pro-drug of 3-BrPA, 3-bromo-pyruvate propyl ester (3-BrOP) was used instead. 3-BrOP showed very high citotoxicity at low dosage (Figure 2C), suggesting that the ability of 3-BrOP to enter the cells and release 3-BrPA via hydrolysation by cellular esterases accounts for the > 10 fold difference in the potency of the two compounds. However, its efficacy was not improved by hypoxia (Table 1A).

**Table 1:** (A) Hypoxia affects the EC50 of glycolysis inhibitors in PEL cells

A					
		BCBL1		HBL6	
Drug	Molecular target	EC50 21% O_2_	EC50 1% O_2_	EC50 21% O_2_	EC50 1% O_2_
2-deoxyglucose	HXK	6.63 ± 2.98 mM	1.51 ± 0.16 mM	5.10 ± 0.68 mM	2.43 ± 0.24 mM
sodium-oxamate	LDH-A	76.40 ± 2.42 mM	44.76 ± 4.33 mM	62.78 ± 3.64 mM	54.82 ± 3.21 mM
lonidamine	HXK	N.I. at 100 μM	N.I. at 100 μM	N.I. at 100 μM	N.I. at 100 μM
3-bromopyruvate	HXK, GAPDH	175.74 ± 11.14 μM	232.39 ± 11.14 μM		
3-bromo-pyruvate propyl ester	HXK, GAPDH	34.39 ± 6.13 μM	38.13 ± 3.08 μM	43.86 ± 1.93 μM	52.61 ± 2.28 μM
dichloroacetate	PDK	25.78 ± 1.29 μM	25.29 ± 0.70 μM	25.31 ± 0.92 μM	24.45 ± 2.88 μM

B			
		BCBL1	
Drug	Molecular target	EC50 21% O_2_	EC50 1% O_2_
PF-04691502	PI3K/mTOR	1.44 ± 0.4 μM	1.06 ± 0.2 μM
Akti 1/2	Akt	1.32 ± 0.15 μM	0.92 ± 0.11 μM

C			
		BCBL1	
Drug 1	Drug 2	C.I. (50;75;90) 21% O_2_	C.I. (50;75;90) 1% O_2_
PF-04691502	2-deoxyglucose	0.34;0.36;0.39	0.67;0.46;0.49
Akti 1/2	2-deoxyglucose	0.37;0.40;0.29	0.68;0.53;0.60

**Figure 4:**
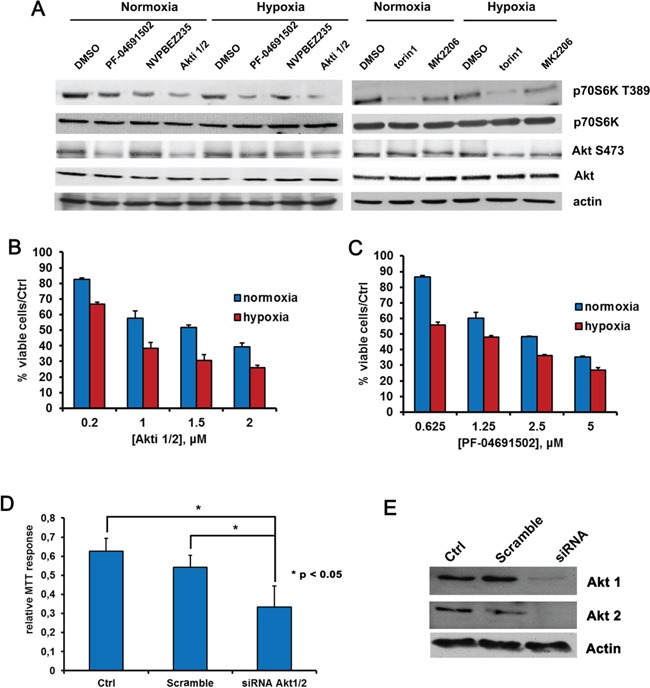
Hypoxia influences BCBL1 cells sensitivity to Akt or PI3K/mTOR inhibition **A.** BCBL1 cells were cultured for 24 hours in hypoxia or normoxia and treated with PF-04691502 0.625 μM, NVPBEZ235 50 nM, Akti1/2 200 nM, torin1 100 nM and MK2206 1 μM. Equal amounts of cellular protein extract were resolved by SDS-PAGE and probed with the indicated antibodies. Equal loading was confirmed by anti-β-actin. Cell viability upon 24 hours treatment with Akti1/2 **B.** or PF-04691502 **C.** was monitored by MTT assay, as described previously. **D.** Cells were transfected with siRNA directed to human Akt1 and 2, or with scramble siRNA, by means of the Amaxa nucleofection system using the nucleofector solution V, then cell viability was detected by MTT. **E.** Equal amounts of cellular protein extracts form samples described in (D) were resolved by SDS-PAGE and probed with anti-Akt1 or anti-Akt2. Equal loading was confirmed by anti-β-actin.

CBL1 and HBL6 cells were treated with increasing concentrations of 2-DG, lonidamine, 3-BrPA, 3-BrOP, DCA, oxamate as described in Figure 2, panels A to E. MTT assays were performed in triplicates, and the EC50 in normoxia (21%) and in hypoxia (1%) was calculated. Mean values and S.D. are indicated. (B) Hypoxia affects the EC50 of PI3K/mTOR and Akt inhibitors in PEL cells. BCBL1 cells were treated for 24 hours with increasing concentrations of Akti 1/2 or PF-04691502, as described in Figure 4 panels B and C respectively. Then MTT assays were performed in triplicates, and the EC50 in normoxia (21%) and in hypoxia (1%) was calculated. Mean values and S.D. are indicated. (C) Combined treatment with 2-DG and PI3K/mTOR or Akt inhibitors displays synergistic cytotoxicity in PEL cells. BCBL1 cells were treated for 24 hours with 2-DG in association with PF-04691502 or with Akti 1/2 at the concentrations reported in Figure 6, panels A and C respectively. The CI in normoxia (21%) and in hypoxia (1%) was calculated with the CalcuSyn algorithm.

### Hypoxia inhibits the mTOR pathway in PEL cells

Cellular responses to hypoxia are known to reduce chemotherapeutic sensitivity through various direct or indirect mechanisms acting on survival pathways [51], such as the PI3K/Akt/mTOR signaling cascade. To get more insights into this issue, PEL cells grown for 24 hours either in normoxia or in hypoxia were profiled by means of reverse phase protein array analysis (RPPA) and Western blotting, with a panel of antibodies recognizing primarily phosphorylated epitopes of the PI3K/Akt/mTOR signaling pathway (Figure 3A–3C). HeLa cells were used as positive control, since in these cells the above pathway is known to be constitutively active [52]. The result not only confirms that the pathway is constitutively activated in all samples but also demonstrates that hypoxia negatively affects it at the level of mTORC1 and its downstream substrate P70S6K (Figure 3B). These results were confirmed by Western blotting (Figure 3C and Supplementary Table S2A). Stabilization and activation of the Hypoxia Inducible Factor 1 (HIF1) was monitored as a readout of hypoxia, assessing the expression of the regulatory subunit HIF1α (Figure 3C and Supplementary Figure S2B–S2C). HIF1 has been demonstrated to be a master regulator of the switch in glucose metabolism from oxidative respiration to aerobic glycolysis [53, 54]. HIF1-mediated hypoxia response and the PI3K/Akt/mTOR pathway have demonstrated an intimate mutual dependence, and can act in an integrated way, even though the modality of the interaction is largely dependent on cell-type and experimental set-up [55]. Opposite to what reported previously in different cell models, in PEL cells forced expression of a constitutively active form of Akt, myrAkt [56], which inactivates TSC1/2 by direct phosphorylation, was not sufficient to stabilize HIF1α in normoxia (Supplementary Figure S2A). On the other hand, mTOR inhibition by rapamycin, which targets mTORC1, and by torin1, which blocks both mTORC1 and 2, decreased hypoxia-induced HIF1α levels (Supplementary Figure S2B), indicating that the mTOR pathway is necessary for the full realization of the HIF-mediated response to hypoxia in these cells.

### Hypoxia alters PEL cells sensitivity to inhibitors of the PI3K/Akt/mTOR pathway

PI3K/Akt/mTOR is a converging hub of both oncogenic and metabolic signaling [57–59]. Recently it has been shown to strongly affect cellular metabolism, favoring and supporting the Warburg phenotype in cancer cells both directly and indirectly: as a target of multiple growth factors, PI3K stimulation can lead to chronic activation of Akt in cancer cells, which in turn can directly increase the expression of glucose transporters and glycolytic enzymes [60–65]. Besides, PI3K can influence cell metabolism indirectly through the stimulation of mTORC1, that in turn controls amino acid metabolism and protein translation [66]. Importantly, the dual PI3K and mTOR inhibitor NVPBEZ235 was previously shown to counteract the glycolytic phenotype of the BCBL1 PEL cell line and to delay tumor progression in a xenograft model of PEL [10]. The high activation of Akt, moreover, has been recently shown to drive glycolysis of leukemic cells [67]. Therefore, we investigated the effect of a panel of drugs targeting either Akt, namely the allosteric inhibitors Akti 1/2 and MK2206, or mTOR, namely torin1, or both PI3K and mTOR, namely the abovementioned NVPBEZ235 and the second generation dual PI3K and mTOR inhibitor PF-04691502, on the glycolytic BCBL1 cell line. All the above drugs inhibited their target efficiently, as demonstrated by the decreased phosphorylation of Akt S473 and P70S6K T389 (Figure 4A and Supplementary Table S2B–S2C), indicating that in PEL cells this pathway is indeed druggable. Furthermore, we assessed that they were all cytotoxic on PEL cells, with an EC50 in the nanomolar or low micromolar range. In particular, given the perturbation of the pathway during hypoxia, which might potentially alter cellular sensitivity to the drugs, we compared the cytotoxic activity of the compounds in normoxia and in hypoxia. It is worth noting that while torin1, MK2206 and NVPBEZ235 effectiveness was barely affected (Supplementary Figure S3A–S3C and Supplementary Table S1), hypoxia did enhance cytotoxicity of both the Akti 1/2 and the PF-04691502 inhibitors (Figure 4B–4C), in good agreement with the significantly lower EC50 values displayed by the same two drugs in hypoxia. The results are summarized in Table 1B and Supplementary Table S1. Next, to ascertain that the effects of the inhibitors were not due to off-target action, the expression of Akt1 and 2 (the Akt isoforms detectable in these cells) was silenced by a specific siRNA directed to a sequence common to Akt 1 and Akt2, and compared to scramble siRNA. As expected, the downregulation of Akt expression dramatically decreased cell viability (Figure 4D).

### Disruption of the PI3K/Akt/mTOR pathway in PEL cells affects glucose metabolism

**Figure 5:**
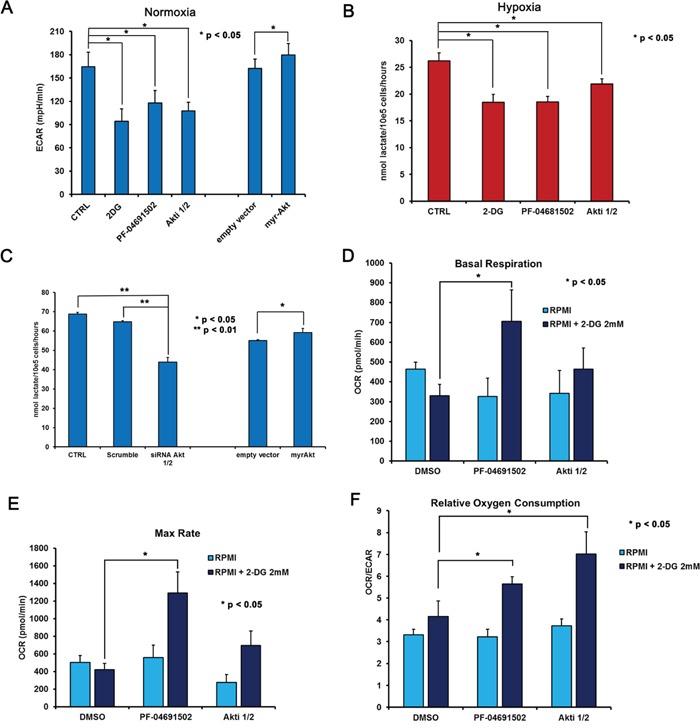
2-DG inhibition of glycolysis combined with Akt and PI3K/mTOR inhibition results in increased oxidative metabolism BCBL1 cells, treated for 24 hours with vehicle (CTRL), 0.625 μM PF-04691502, 200 nM Akti1/2, 1 mM 2-DG as indicated, either in normoxia (A) or in hypoxia (B) Panel **A.** cells were counted and plated at 150.000 cell/well in XF96 culture plates prior to the assay, then ECAR was calculated in control cells, upon addition of 2-DG or PI3K/Akt/mTOR inhibitors for 24 hours, as well as in BCBL1 cells transiently transfected (24 hours) with empty vector or with the constitutively active myrAkt vector. Panel **B.** the level of lactate in the culture medium of BCBL1 grown in hypoxia for 24 hours was measured as described in Methods. The data are expressed as the mean ± S.D. of three different replicates. Panel **C.** BCBL1 cells were transfected either with siRNA to Akt1/2 as in Figure 4D, or with empty vector or myr-Akt as in (A) Then secreted lactate was assayed in the supernatant. Panels **D.** and **E.** represent Basal Respiration and Max Respiratory Capacity, respectively, in cells exposed to vehicle (DMSO), 0.625 μM PF-04691502, 200 nM Akti1/2 alone (pale blue bars) or in the presence of 2 mM 2-DG (dark blue bars). Panel **F.** shows the Relative Oxygen Consumption by the OCR/ECAR ratio, in the same setting as in (D) and (E). Each experiment was performed at least three times. Where indicated, unpaired *t*-Test was performed on each mean value.

We next investigated whether modulation of the pathway by the indicated drugs may be useful to control PEL cell metabolic rewiring. BCBL1 cells were treated as described above, then the glucose-to-lactate flux was calculated, based on the ECAR of the culture media. Remarkably, all treatments caused a reduction of ECAR, i.e. of glycolysis, to an extent close to that obtained with 2 mM 2-DG (Figure 5A), with the exception of MK2206 (Supplementary Figure S4A). In agreement with this finding, we observed that, although glycolysis in basal condition is already very high in these cells, an additional increase of Akt activity through forced expression of its constitutively active form, led to a small but significant extra boost of ECAR (Figure 5A). Moreover, when cells were grown in low oxygen, all treatments significantly counteracted the production of lactate (*p* < 0.05) (Figure 5B). Similar results were obtained by means of silencing Akt with specific siRNA (Figure 5C). We concluded therefore that the effects described above, triggered by addition of these drugs to BCBL1 cells, are indeed due to the inhibition of the activity of their target kinases.

**Figure 6:**
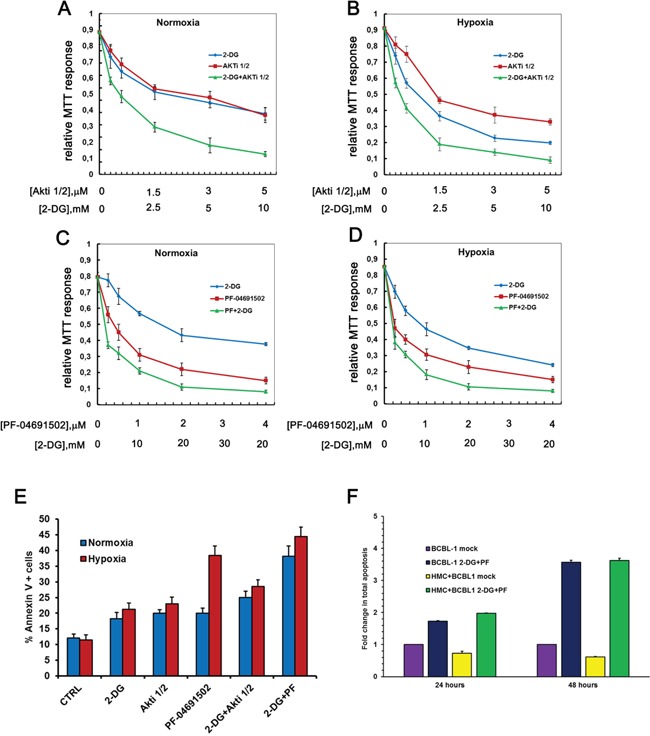
Hypoxia strenghtens the cytotoxicity of the drug treatment BCBL-1 cells were grown in normoxia or in hypoxia, treated with 2-DG alone or in combination with Akti1/2 A, B. or PF-04691502 C, D. at the indicated concentrations, for 24 hours. Graphs A to D show the MTT response relative to controls. CI was calculated with the CalcuSyn algorithm. **E.** From the same experimental setting, total apoptosis of cells treated with 2 mM 2-DG with or without 625 nM PF-04691502 or 1 μM Akti1/2 was assessed by Annexin V staining. **F.** BCBL1 cells were co-cultured for 24 or 48 hours with HMC in a medium additioned with vehicle (mock), with 625 nM PF-04691502 or with 1 μM Akti1/2. Total apoptosis was calculated as the mean percentage of Annexin V positive BCBL1 cells in each condition, as indicated.

This observation confirms the involvement of the PI3K cascade in the regulation of glucose metabolism in PEL cells. As a consequence of the reduced glucose utilization, also OCR appears slightly inhibited in both basal condition (Figure 5D) as well as upon FCCP-stimulated maximal respiration (Figure 5E). Notably the OCR/ECAR ratio remains mostly unchanged, suggesting that inhibition of the PI3K cascade alone does not revert the Warburg effect characterizing this cell line (Figure 5F). Quite unexpectedly, however, we found that in combination with 2-DG these compounds increased both basal and max respiration rate (Figure 5D–5E). Accordingly, 2-DG combined with PF-04691502 or with Akti 1/2 resulted in a significant (*p* < 0.05) boost of the OCR/ECAR ratio (Figure 5F). In particular, the combination of 2-DG with PF-04691502 as well as with Akti 1/2 was characterized by high oxygen consumption, and resulted in a significant (*p* < 0.05) shift from aerobic glycolysis towards a more oxidative respiration (Figure 5E). We asked whether such a shift might render cancer cells more susceptible to induction of apoptosis. Therefore we next tested the cytotoxicity of these drug combinations on PEL cells by MTT assay. The association with 2-DG clearly drops cell viability (Figure 6A–6E), with a concentration-dependent effect, as indicated by the combination index (CI) values (Table 1C), calculated according to Chou&Talalay [68]. The results point to a strong synergism (CI < 0.5) of 2-DG in association with Akti 1/2 or with PF-04691502, both in normoxia and in hypoxia (Table 1C). In particular, hypoxia further diminishes cell viability by these combinations, which thus might prove useful as a novel therapeutic approach for PEL. However, because these results were obtained by means of a metabolic assay based on mitochondrial activity, which might be affected by the drugs, apoptosis triggered by single or combined treatments was assessed by Annexin V staining. The result demonstrates that 2-DG indeed potentiates the effect of both Akti 1/2 and, to a greater extent, PF-04691502. Importantly, it also shows that a low oxygen environment further augments the number of Annexin V positive cells (Figure 6E), strengthening the concept that this type of drug association should be taken into account as a novel approach in PEL therapy.

Due to the recently demonstrated shielding effect of the mesothelium on lymphoma progression [69], we then asked whether the mesothelial microenvironment protects against 2-DG+PF-04691502-facilitated apoptosis. To mimic the physiological microenvironment, BCBL1 cells were co-cultured with primary human mesothelial cells (HMC) for 48 hours, a condition sufficient to highlight the pro-survival effect of HMC on BCBL1 cells (Figure 6F). Then cells cultures were subjected to treatment with DMSO vehicle (mock) or with a combination of 2-DG and PF-04691502 for 24 or 48 hours. Total apoptosis of the BCBL1 population was determined by means of Annexin V staining. While HMC co-culture proved able to protect PEL cells from apoptosis in basal condition, it is clear that it is not sufficient to abrogate the pro-apoptotic effect of the drugs nor to diminish its efficacy (Figure 6F and Supplementary Figure S5A–S5B).

**Figure 7:**
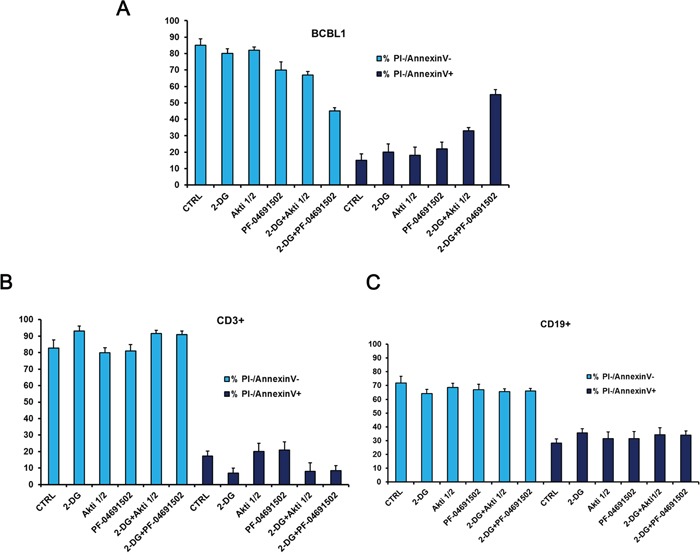
Glycolysis inhibition combined with Akt or PI3K/mTOR inactivation displays low cytotoxicity to primary lymphocytes from healthy donors BCBL1 and peripheral blood mononuclear cells (PBMCs) were treated for 24 hours as indicated, then stained with PI and Annexin V followed by flow cytometry analysis. PBMCs from healthy donors were labeled with anti-CD3+ or with anti-CD19+ to detect T-lymphocytes and B-lymphocytes respectively. The graphs show the percentage of viable (double negative PI/Annexin V) and apoptotic (PI negative/Annexin V positive) BCBL1 cells **A.** CD3+ cells **B.** or CD19+ cells **C.** The data are expressed as the mean ± S.D. of three different replicates.

Finally to investigate the toxicity of the proposed drug combinations on normal cells, BCBL1 cells were grown for 24 hours in the presence of 2-DG ± PF-04691502 or Akti 1/2, and the viability, assessed by Annexin V/PI staining, was compared to that of normal human B (CD19+) and T (CD3+) lymphocytes from healthy donors. It is very interesting to note that in both cases drugs appear to exert little toxicity on normal human lymphocytes, compared to PEL cells (Figure 7B–7D), suggesting some degree of specificity towards PEL cells.

## DISCUSSION

Remodeling of energy metabolism from oxidative phosphorylation to oxygen-independent aerobic glycolysis is becoming a sensible area for drug targeting. PEL is a B-cell NHL characterized by a very poor prognosis, with a median survival of six months after the diagnosis and no standard therapy [1–4]. In agreement with a previous report [26], we detail here that PEL cells preferentially channel glucose towards lactate production even in the presence of oxygen, and thus display a glycolytic metabolism. A large body of evidence has shown that the glycolytic phenotype in cancer cells is due to active glycolysis prevailing over mitochondrial respiration rather than to defects in mitochondrial function [70]. The observation that proliferation and tumorigenicity of cancer cells can be inhibited blocking glycolysis, however, suggests that enhanced oxidative respiration is not sufficient to meet the requirement of cancer growth and that glycolysis is a target of cancer therapy [71–73]. In our work, we confirm that BCBL1 cells maintain the ability to produce ATP by mitochondrial respiration, and that inhibition of glycolysis by 2-DG is toxic to these cells. We also demonstrate that 2-DG as well as other glycolysis inhibitors such as oxamate are particularly effective against PEL in low-oxygen settings, a condition that mimics the highly hypoxic environment of body cavities [28–29]. This indicates that in hypoxia PEL cells are even more reliant on glycolysis for their energy requirements and thus intrinsically more exposed to its inhibition. Although its potential as monotherapy is poor, in spite of the promising results *in vitro* and in animal models as well as of its excellent safety profile as anti-cancer drug, 2-DG has been shown to enhance the efficacy of many drugs when administered in combination [74, 75].

The PI3K/Akt/mTOR signaling can favor and support the Warburg phenotype in cancer cells both directly and indirectly. PI3K stimulation by multiple growth factors can lead to chronic activation of Akt in cancer cells, which directly increases the expression of glucose transporters and glycolytic enzymes, and in turn glucose metabolism in cancer cells versus normal cells [61, 62]. In particular, Akt has been shown to regulate HXK expression, activity, and mitochondrial interaction [76], as well as the localization of the glucose transporter GLUT1 to the plasma membrane [60]. In addition, phosphorylation of phosphofructokinase-2 by Akt [77] drives allosteric activation of PFK1. Akt activity correlates to the degree of a high level of aerobic glycolysis without increasing oxygen consumption [62], and has been recently shown to drive glycolysis of leukemic cells [67]. In previous studies, the efficacy of the mTOR inhibitor rapamycin in PEL cells in culture and in a xenograft PEL model was limited by the rapid emergence of drug resistance [9], whereas dual targeting of PI3K and mTOR by the inhibitor NVP-BEZ235 gave better results [10]. In this study the animal PEL model was obtained by subcutaneous injection of PEL cells, though, rather than by peritoneal engraftment, which would reproduce the *in vivo* pathology more closely. Taking the view that alteration of intracellular signaling through the PI3K pathway is deemed crucial to achieve a cancer metabolic phenotype through the Warburg effect, our study highlights the previously unreported observation that pathway-specific inhibitors targeting either PI3K/mTOR or Akt counteract lactate production to an extent similar to that obtained by 2-DG, as indicated by the shift of the ECAR. Remarkably, in these cells PF-04691502 is able not only to significantly decrease the extracellular acidification but also to deeply enhance the mitochondrial respiration rates, thus switching aerobic glycolysis towards a more oxidative metabolism. As expected, all drugs dramatically decrease cell viability. Most importantly, both effects are enhanced by association with 2-DG, pointing to a strong synergistic action, both in normoxia and in hypoxia. Although the rationale of this synergy is not completely understood, it is worth remembering that 2-DG also increases oxidative stress, affects protein glycosylation, and therefore causes aberrant GlcNAcylation of proteins and accumulation of misfolded proteins in the endoplasmic reticulum, leading to autophagy and ER stress response [78, 79]. This last effect may be particularly relevant to our work, as it has been recently demonstrated that Akt inhibition potentiates toxicity induced by 2-DG through ER stress response in acute lymphoblastic leukemia by enhancing the unfolded protein response. [80]. Moreover, it has been shown recently that 2DG can also modify the expression profile of cancer cells [81]. In particular, it was claimed to induce the transient expression of p21 and a stable expression of p53, together with cell cycle arrest at G0/G1 phase and apoptosis through the intrinsic, mitochondrial pathway in colorectal cancer cells [81]. Interestingly these effects were totally independent of its inhibitory effect on either hexokinase or ATP levels. All together these studies provide key insights about processes, essential for the biological properties of 2-DG, that act beyond the metabolic block, and might prompt cancer cells to the pro-apoptotic action of the drugs used in our study.

Moreover, it is particularly important that the pro-apoptotic function of this drug association also prevails over the shielding action of the HMC in the HMC-BCBL1 co-culture performed to mimic the PEL microenvironment [69]. Finally, it is worth remembering that we have shown here that the above drug associations exert very low toxicity in normal human B and T lymphocytes, compared to PEL cells, suggesting some degree of specificity towards cancer cells. Thus, all together, our results open a new therapeutic approach for PEL. Notably, because the above combinations are highly effective also under normoxic conditions, they could be extended to other B-NHL characterized by a glycolytic phenotype.

## MATERIALS AND METHODS

### Chemicals and reagents

Akti 1/2 was purchased from Calbiochem, rapamycin, 2-deoxy-D-glucose (2-DG), dichloroacetate (DCA) and 3-bromo pyruvate (3-BrPA), were from Sigma-Aldrich, PF-04691502, torin1 and MK2206 were from Selleckchem, lonidamine from LTK Laboratories and NVPBEZ235 were from Axon Medchem. A derivative of 3-BrPA, 3-bromo-2-oxopropionate-1-propyl ester (3-BrOP), was synthesized by esterification of 3-BrPA with 1-propanol (Sigma-Aldrich) as previously described [82]. siRNA to Akt1 and 2 were from Origine (Rockville, MD, USA).

### Cell culture and transfection

PEL cell lines BCBL1 and HBL6 were maintained in RPMI1640 supplemented with 20% heat-inactivated fetal bovine serum (FBS), 100 U/ml penicillin, 100 U/ml 12 streptomycin, 2 mM L-glutamine. Both cell lines are KSHV-positive; HBL6 are also EBV-positive. Cells were grown under either normoxia (21% O_2_, 5% CO_2_) or hypoxia (1% O_2_, 5% CO_2_) at 37°C for the indicated times and drug concentrations. Low O_2_ tension was obtained using either a Hypoxia Chamber (StemCell Technologies) or the INVIVO_200_ hypoxia workstation (Ruskinn Technology Ltd., UK). Transfection of BCBL1 cells was performed using the Amaxa nucleofection system (Amaxa, Cologne, Germany). Briefly, 5–10 × 10^6^ cells were resuspended in nucleofector solution V prior to addition of DNA (CMV6-HA-myrAkt, CMV6 empty vector, siRNA to Akt 1 and 2 or scramble siRNA), then transferred to an Amaxa-certified cuvette and transfected using program T-001. Cells were examined for the expression of transfected genes 24 to 48 hours post transfection.

### Western blotting and RPPA analysis

Samples were extracted in TPER reagent (Pierce, Rockford, IL, USA). For Western blotting, 30 μg of total lysates were separated by 10% SDS-polyacrylamide gel electrophoresis and transferred to PVDF membrane (Millipore) as previously described [83]. Array assembly, printing, staining and analysis were performed as described [84–86]. Antibodies against phosphorylated epitopes were from Cell Signaling Technologies. Anti-β-actin was from Sigma Aldrich. Anti-HIF1α was from BD Biosciences. Differences in basal phosphorylation of treated samples were compared by calculating the ratio of each sample's SI (signal intensity) divided by vehicle-(DMSO) or drug-treated sample's SI. To determine the significance of the difference in mean of treated samples we used a Paired student's *t* test with a *P*-values = 0.05.

### MTT assay

Viability of PEL cells treated with various inhibitors or the appropriate vehicle as a negative control was determined by the 3-(4,5-dimethylthiazol-2-yl)-2,5-diphenyltetrazolium bromide (MTT)-based colorimetric assay as previously described [87]. Where indicated, effective dose 50s and combination index were calculated following the Chou-Talalay method [68] and the softwares GrafIt and CompuSyn (ComboSyn InC.)

### Lactate assay

Cells were rinsed in fresh media and grown for 6 or 24 hours in the presence or absence of inhibitors as indicated. Media samples were then collected, flash-frozen in liquid nitrogen and immediately stored at −80°C until the time of the assay. Lactate levels in media were measured using a Lactate Assay Kit (Eton Bioscience Inc.) according to manufacturer instructions.

### Flow cytometry analysis

For cell cycle analysis, 5 × 10^5^ cells were centrifuged at 1200 rpm for 8 minutes, washed with PBS and fixed with cold 70% EtOH for 2 hours, and incubated with 25 μg (1 μl) of RNAse (Sigma Aldrich, 25 μg/μl) for 1 hour at 37°C. PI (25 μl, 5 mg/ml, Sigma Aldrich) was added fresh prior to analysis. For detection of apoptosis, 1 × 10^6^ cells were washed twice with PBS and stained with Annexin V–FITC and PI in 1X binding buffer (10 mM HEPES, pH 7.4, 140 mM NaOH, 2.5 mM CaCl2) for 15 min at room temperature in the dark. Both early apoptotic (Annexin V+, PI-) and late (Annexin V+ and PI+) apoptotic cells were included in cell death determinations. Annexin V- and PI-negative cells are viable cells. The analysis was performed on a FACSCalibur (BD Bioscences, NJ, USA).

### BCBL1-mesothelial cells co-culture

Primary human mesothelial cells (HMC) were isolated and cultured as previously reported [69]. To evaluate the activity of 2-DG and PF on BCBL1 cells in a context mimicking the intracavitary environment, 2 × 10^5^ BCBL1 cells were co-cultured with subconfluent cells first passage HMC in 6-well plates with or without drug treatment for 48 hours. Total apoptosis of BCBL1 cells was quantified by flow cytometry after Annexin V staining (Annexin-V-FLUOS Staining Kit, Roche Diagnostics, Mannheim, Germany). Data were analyzed using the Kaluza Flow Analysis software (Beckman Coulter). SD of the ratio is calculated according to the theory of error propagation, as previously reported [88].

### RT-PCR

Total RNA extraction was as described [83], using the Aurum Total RNA Fatty and Fibrous Tissue kit (Bio-Rad, Hercules CA, USA) according to manufacturer's instructions. Genomic DNA was removed by DNase treatment. MCT-1 and isoform-specific PKM1 (NM_182470) and PKM2 (NM_002654) forward and reverse primers were as follows: 5-TTTCTTTGCGGCTTCCGTTGTTG-3; antisense, 5-TCAATTTACCCTTCAGCCCCATGG for MCT-1; 5′-CTGAGGCAGCCATGTTCC-3′ and 5′-CCATGAGGTCTGTGGAGTG-3′ for PKM1; 5′-ACTTGGTGAGGACGATTATG-3′ and 5′-CTGCCATCTACCACTTGC-3′ for PKM2. PCR primers were designed using Beacon Designer 2.06 (Premier Biosoft International). Real-time amplifications, using SYBR Green detection chemistry, were run in triplicate on 96-well reaction plates with the CFX96 machine (Bio-Rad).

### XF bioenergetic analysis

Oxygen consumption and extracellular acidification rates in PEL cells were measured using the Seahorse XF96 instrument (Seahorse Biosciences, North Billerica, MA) according to the manufacturer's protocols. 24 hours after treatment, cells were seeded in a poly-lysine coated XF96 microplate at the density of 150000 cells per well, for 60 minutes in a 37°C non-CO2 incubator, while sensor cartridges were calibrated prior to the start of an assay. After adhesion, medium was changed with 175 μl unbuffered XF assay media at pH 7.4 supplemented with 5.5 mM glucose (Sigma), 1 mM sodium pyruvate and 1 mM Glutamine. Respiration was measured in four blocks of three for 3 min. The first block measured the basal respiration rate. Next 2 μM oligomycin was added to inhibit complex V (second block). Then 0.3 μM carbonyl cyanide 4-(trifluoromethoxy)-phenylhydrazone (FCCP, Seahorse Biosciences, North Billerica, MA) was added to uncouple respiration (third block). Finally, 1 μM antimycin A and 1 μM Rotenone was added to inhibit complex III (forth block). All reagents are from Seahorse Biosciences, North Billerica, MA. Immediately after finishing the measurements, cells were washed with phosphate-buffered saline, fixed with 4% paraphormaldehyde and stained with 0.1% crystal violet (1 mol/l acetic acid) and absorbance at 595 nm was measured as index of cell amount.

### Measurement of cellular ATP content

Intracellular ATP was measured by bioluminescence using a luciferin-luciferase system (ATP bioluminescent assay kit CLS II; Roche) as described previously [89]. The amount of ATP measured was referred to the protein content, determined by the method of Lowry [90], and expressed as nmol/mg protein.

### Statistical Analysis

The data were presented as mean ± standard deviation, with at least three replicates used for each data point. Unless otherwise indicated, a paired Student's *t* test was performed for each experimental group to assess the statistical significance against respective controls.

## SUPPLEMENTARY FIGURES AND TABLES




