# Correction: Reversal of the glycolytic phenotype of primary effusion lymphoma cells by combined targeting of cellular metabolism and PI3K/Akt/ mTOR signaling

**DOI:** 10.18632/oncotarget.28898

**Published:** 2026-08-14

**Authors:** Laura Mediani, Federica Gibellini, Jessika Bertacchini, Chiara Frasson, Raffaella Bosco, Benedetta Accordi, Giuseppe Basso, Massimo Bonora, Maria Luisa Calabrò, Adriana Mattiolo, Gianluca Sgarbi, Alessandra Baracca, Paolo Pinton, Giovanni Riva, Enrico Rampazzo, Luca Petrizza, Luca Prodi, Daniela Milani, Mario Luppi, Leonardo Potenza, Anto De Pol, Lucio Cocco, Silvano Capitani, Sandra Marmiroli

**Affiliations:** ^1^Department of Surgery, Medicine, Dentistry and Morphology, University of Modena and Reggio Emilia, Modena, Italy; ^2^Department of Woman’s and Child’s Health and Institute of Pediatric Research - Città della Speranza Foundation, University of Padova, Padova, Italy; ^3^Department of Morphology, Surgery and Experimental Medicine Section of Pathology, Oncology and Experimental Biology, University of Ferrara, Ferrara, Italy; ^4^Immunology and Molecular Oncology, Veneto Institute of Oncology, IOV IRCCS, Padova, Italy; ^5^Department of Biomedical and NeuroMotor Sciences, University of Bologna, Bologna, Italy; ^6^Department of Medical and Surgical Sciences, Section of Hematology, University of Modena and Reggio Emilia, AOU Policlinico, Modena, Italy; ^7^Department of Chemistry, University of Bologna, Bologna, Italy; ^8^Department of Morphology, Surgery and Experimental Medicine, Section of Anatomy and Histology and LTTA Center, University of Ferrara, Ferrara, Italy; ^*^These authors have contributed equally to this work

**This article has been corrected:** It was noted that in Figure 3C the western blot images for P70S6K and Gsk3b proteins in BCBL1 cells appeared very similar. The authors have confirmed that the image for P70S6K is correct. The authors provided new western blot images of HBL6 and BCBL1 cells grown in 21% or 1% O_2_, probed with anti-Gsk3b and prepared using lysates from the original experiments. No significant differences in the levels of total or phosphorylated Gsk3b were observed between the two culture conditions. These results align with the RPPA analysis data in Figure 3B, which showed similar Gsk3b protein levels in BCBL1 cells under both normoxia and hypoxia. All authors agree with this correction, which does not change the conclusions of the article. New Figure 3 is shown below.

Original article: Oncotarget. 2016; 7:5521–5537. 5521-5537. https://doi.org/10.18632/oncotarget.6315

**Figure 3:**
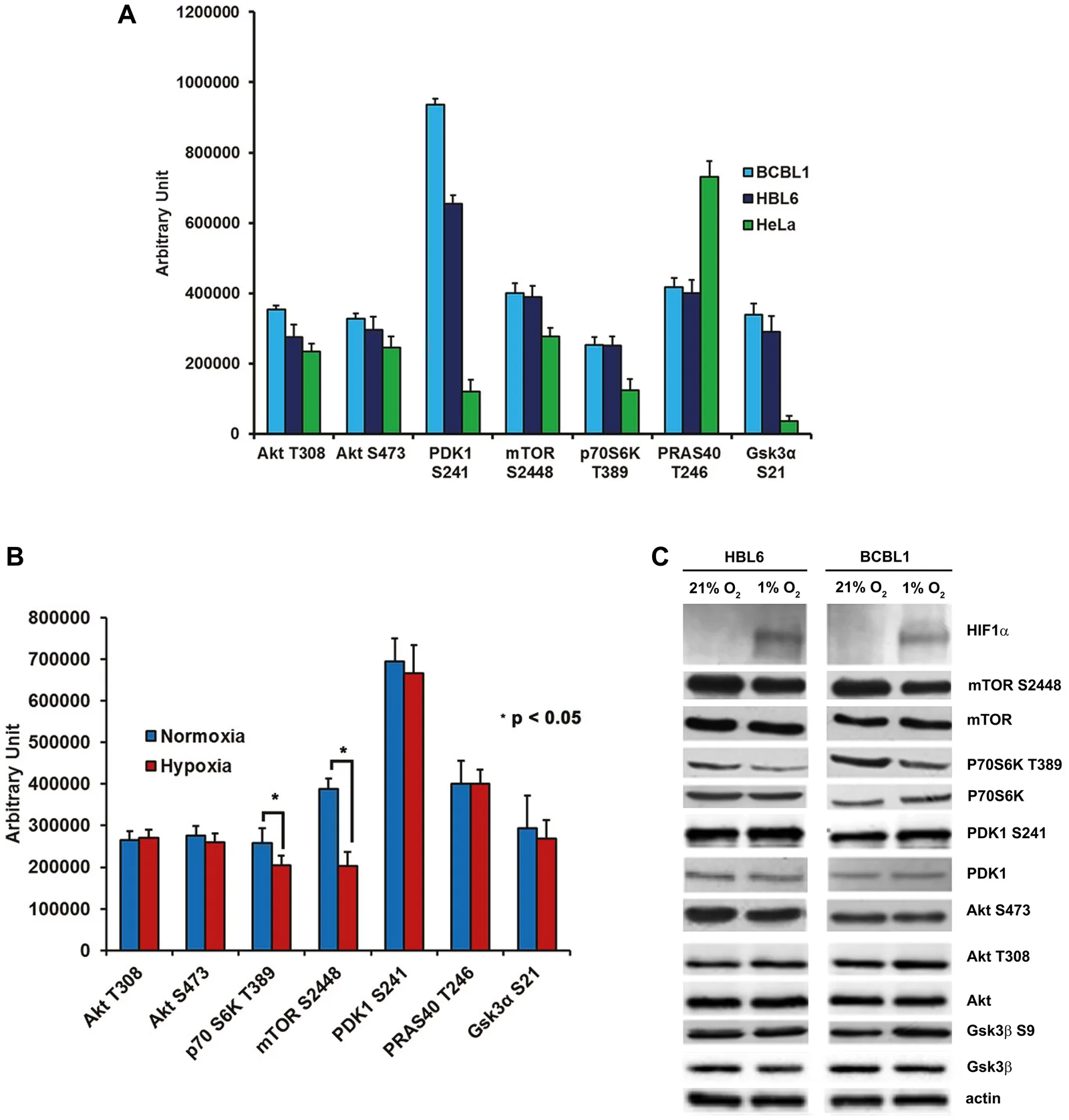
Hypoxia affects the PI3K/Akt/mTOR pathway at the mTORC1 level. **A**. Activation of the PI3K/Akt/mTOR pathway was analyzed by RPPA in BCBL1 and HBL6 cells, and phosphorylation of key molecules was compared with HeLa cells. **B**. RPPA comparison of the phosphorylation status of the indicated signaling molecules in BCBL1 cells grown in normoxia or in hypoxia. **C**. Equal amounts of cellular protein extracts from (B) were resolved by SDS-PAGE and probed with the indicated antibodies. PDK1 S241, Akt T308, Akt S473 are a readout of Akt downstream signaling activation. P70S6K T389 is a readout of mTOR activation. HIF1α was is a readout of hypoxia. Equal loading was confi rmed by anti-β-actin.

