# Supplementary material for: Reversal of the glycolytic phenotype of primary effusion lymphoma cells by combined targeting of cellular metabolism and PI3K/Akt/ mTOR signaling
Source: Oncotarget. 2015 Nov 6;7(5):5521–37. doi: 10.18632/oncotarget.6315 (PMC4868703; doi:10.18632/oncotarget.6315)
Supplement: Supplementary file 1 [file oncotarget-07-5521-s001.pdf]

SUPPLEMENTARY FIGURES

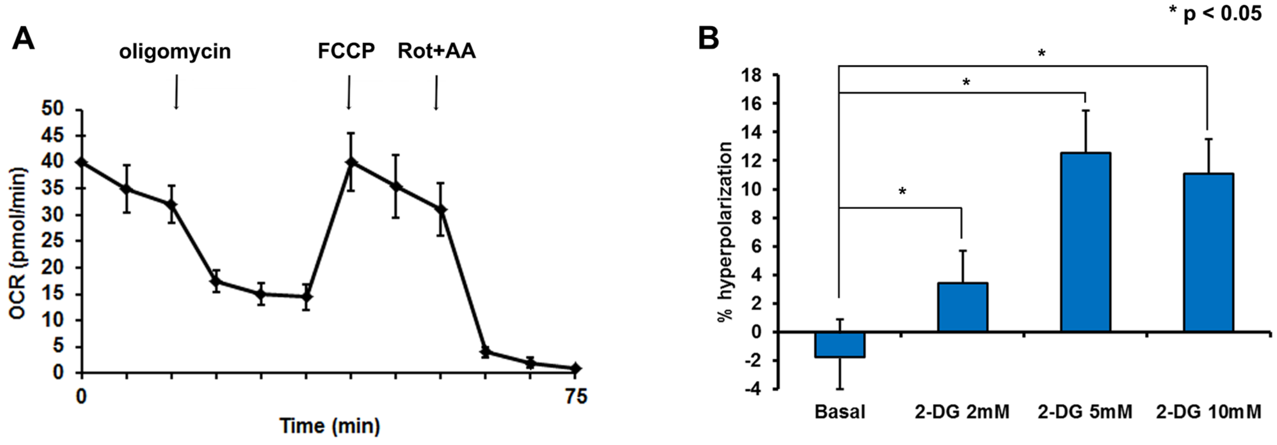

Supplementary Figure S1: Mitochondrial respiration in PEL cells. A. The oxygen consumption rate was analyzed in real time by the XF96 analyzer in PEL cells over 75 minutes. Baseline cellular oxygen consumption is measured, from which basal respiration is derived by subtracting non-mitochondrial respiration. Next oligomycin is added, and the resulting OCR is used to derive ATP-linked respiration and proton leak respiration. Next FCCP is injected, and maximal respiratory capacity is calculated by subtracting non-mitochondrial respiration from the FCCP OCR. Addition of a mix of Antimycin A and rotenone reveals the non-mitochondrial respiration. The mitochondrial reserve capacity is calculated by subtracting basal respiration from maximal respiratory capacity. **B.** The mitochondrial membrane potential was analyzed with the potentiometric dye JC-1 in response to Oligomycin. The block of proton leak through the channel of F1/FO ATP synthase causes a transient mitochondrial hyperpolarization, directly proportional to the proton leak. In BCBL1 hyperpolarization was negligible in basal condition, but increased in a concentration-dependent manner upon addition of 2-DG. Though indirect, this observation indicates that in BCBL1 cells 2-DG enhances ATP production by mitochondria. The experiment was performed three times. Results are represented as mean value ± SD.

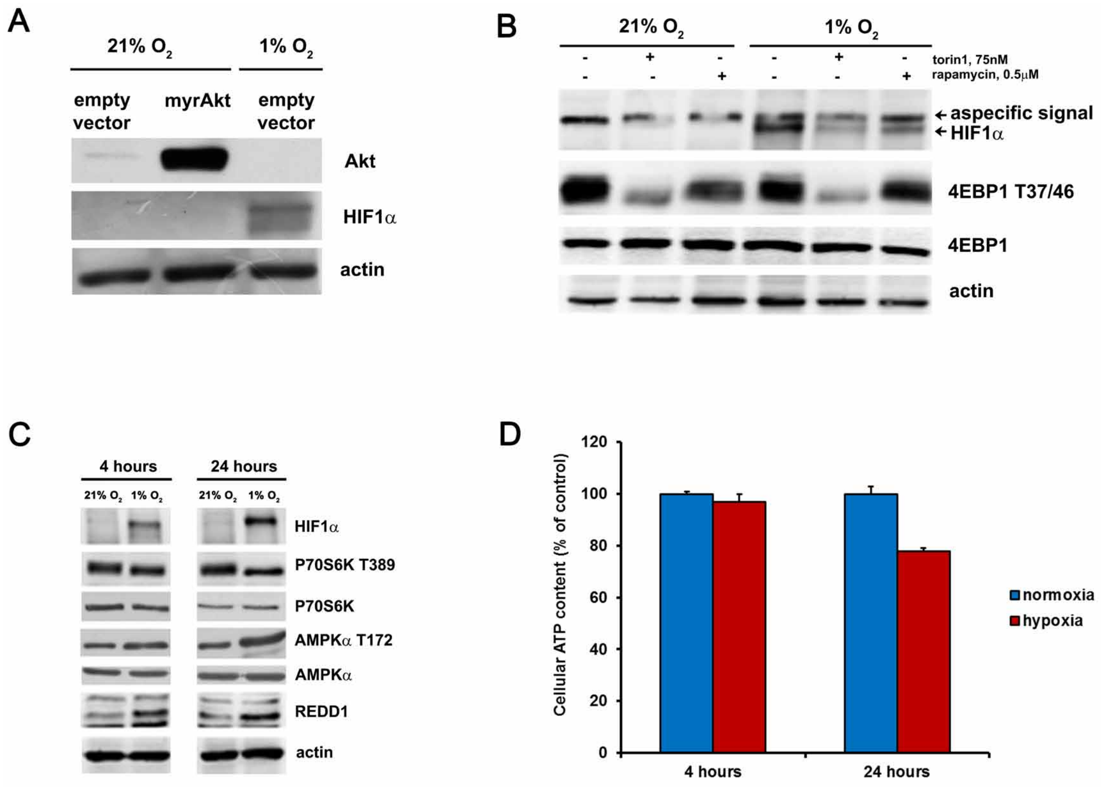

Supplementary Figure S2: Hypoxia affects mTORC1 through REDD1 and AMPK. A. BCBL1 cells were transfected with empty vector or with a constitutively active form of Akt (myrAkt) and grown in normoxia (21% O_2); overexpression of Akt was confirmed by Western blotting. Equal amounts of a lysate from BCBL1 cells cultured in hypoxia for 24 hours (1% O_2) were loaded as positive control of HIF1α induction by hypoxia. Equal loading was confirmed by anti-β-actin. **B.** BCBL1 cells were cultured in normoxia or hypoxia for 24 hours, treated or not with the specific mTOR inhibitors torin1 and rapamycin. Equal amounts of cellular protein extracts were resolved by SDS-PAGE and probed with the indicated antibodies. 4EBP1 T37/46 is a readout of mTOR activation. Equal loading was confirmed by anti-β-actin. **C.** Cells were cultured in normoxia or hypoxia for either 4 or 24 hours. Then activation of the PI3K/Akt/mTOR pathway was analyzed by Western blotting with the indicated antibodies. PDK1 S241, Akt T308, Akt S473 are a readout of Akt downstream signaling activation. P70S6K T389 is a readout of mTOR activation. Equal loading was confirmed by anti-β-actin. **D.** Intracellular ATP was measured in samples from (C) by bioluminescence using a luciferin-luciferase system (ATP bioluminescent assay kit CLS II; Roche) following the manufacturer instructions. The amount of ATP measured was referred to the protein content, and expressed as nmol/mg protein. The assay was performed at least three times. Results are represented as mean value ± SD. It is recognized that low oxygen can affect Akt activity, but the type and extension of the effect depends on the cell type and the severity and duration of hypoxia [51]. Then again, it has been suggested that in some models Akt might influence cellular metabolism *via* HIF1 in normoxia, in response to oncogenic stress. Opposite to the expectation, however, we found that in PEL cells Akt is not sufficient for HIF1 activation in normoxia, regardless of the constitutive activation of the pathway (Supplementary Figure S2A). Nonetheless, similar to other cell models, we observe that hypoxia downregulates mTOR activity (Supplementary Figure S2B and S2C) [52]. Additionally, in agreement with the reported role of mTORC1 to enhance translation of HIF1α [67], in our model pharmacological inactivation of mTOR abrogates almost completely HIF1α upregulation by hypoxia (Supplementary Figure S2B). HIF1-mediated hypoxia response and the PI3K/Akt/mTOR pathway have demonstrated an intimate mutual dependence, and can act in an integrated way, even though the modality of the interaction is largely dependent on cell-type and experimental set-up [51]. Our findings suggest that the downregulation of mTOR signaling is likely mediated through the induction of the HIF1 effector small protein regulated in development and DNA damage responses 1, REDD1 [52], in the early stage of hypoxia (4 hours) (Supplementary Figure S2C). At a later stage (24 hours) an additional contribution by AMPK [53] is conceivable in these cells (Supplementary Figure S2C), as the intracellular ATP starts to decrease (Supplementary Figure S2D). Both REDD1 and AMPK are likely to act through their common target, and mTOR negative regulator, TSC1/2 [52,54].

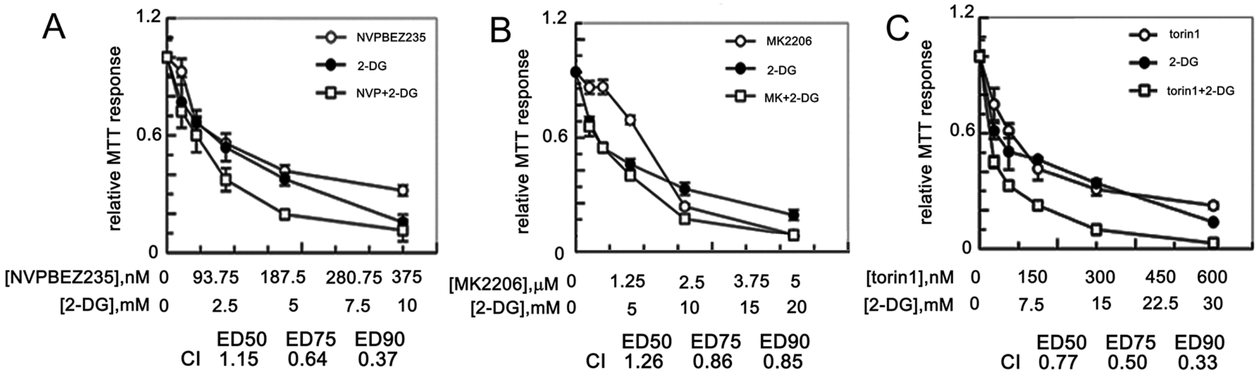

Supplementary Figure S3: Cytotoxicity of 2-DG and PI3K pathway inhibitors to BCBL-1 cells. BCBL-1 cells were grown in normoxia, treated with 2-DG alone or in combination with NVPBEZ235 A. with MK22006 C. or with torin1 at the indicated concentrations, for 24 hours. Graphs A to C show the MTT response relative to controls. CI was calculated with the CalcuSyn algorithm.

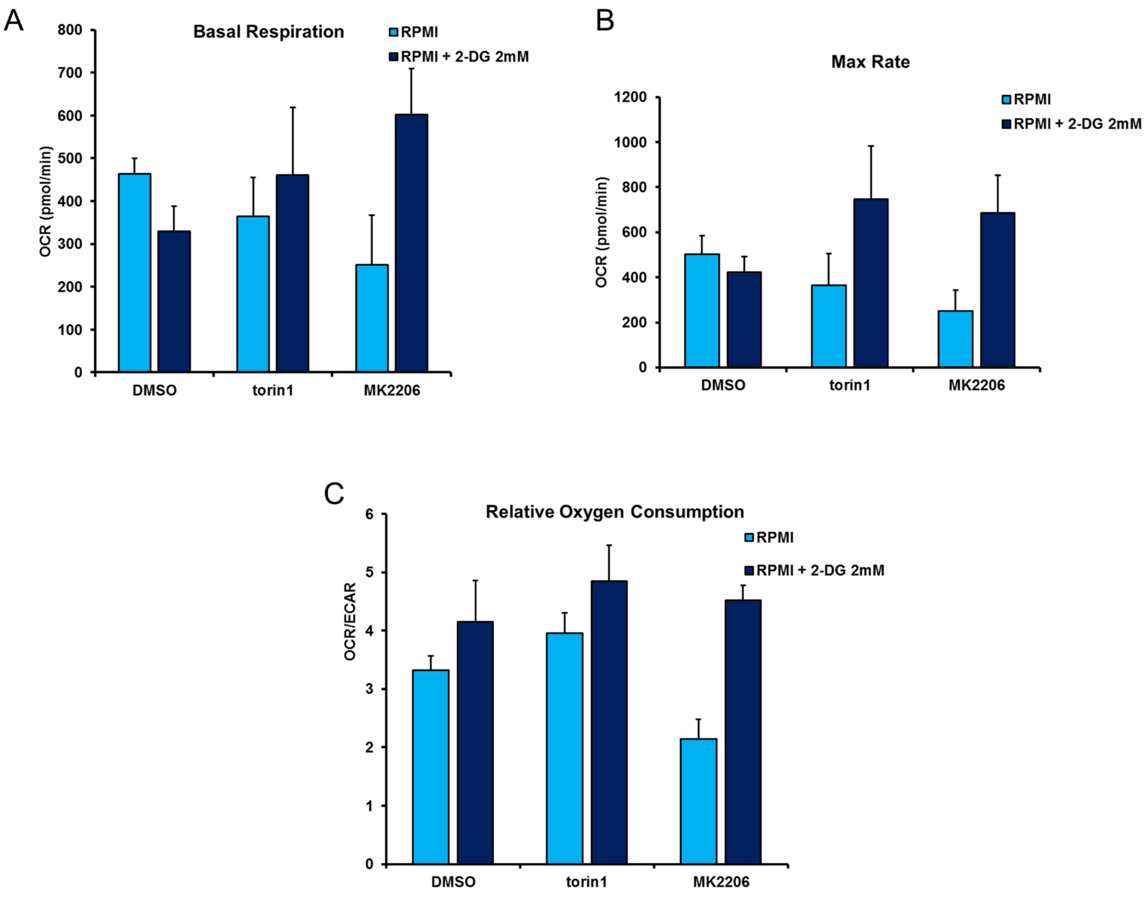

Supplementary Figure S4: PI3K pathway inhibitors affetcts the glycolytic metabolism of BCBL-1 cells. BCBL1 cells were cultured for 24 hours in normoxia. Cells were counted and plated at 150.000 cell/well in XF96 culture plates prior to the assay. Panels **A.** and **B.** represent Basal Respiration and Max Respiratory Capacity, respectively, in cells exposed to either vehicle (DMSO), torin1 100 nM or MK2206 1 µM, alone (pale blue bars) or additioned of 2-DG 2 mM (dark blue bars). Panel **C.** shows the Relative Oxygen Consumption by the OCR/ECAR ratio, in the same setting as in A and B. Each experiment was performed at least three times. Results are represented as mean value ± SD.

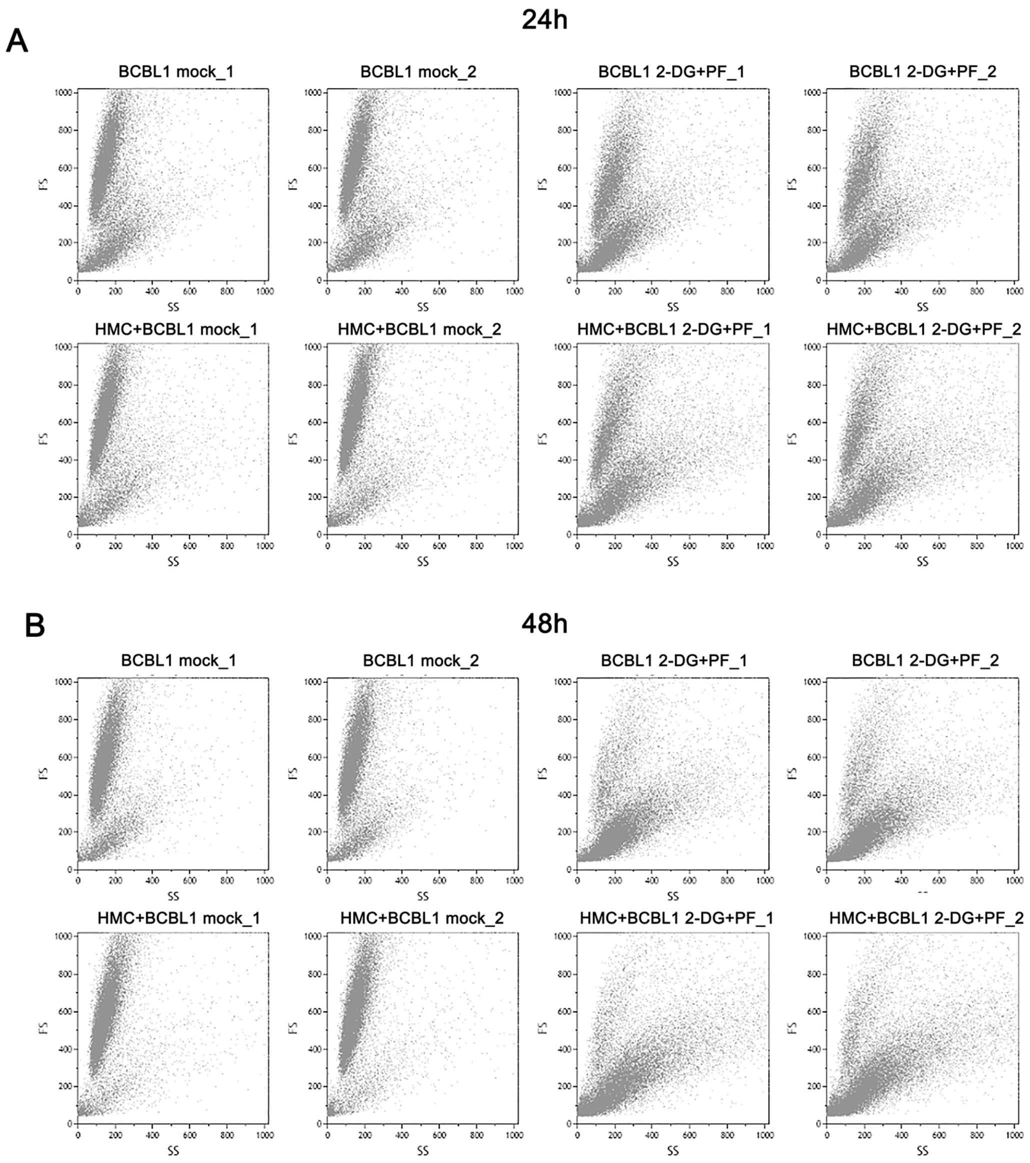

Supplementary Figure S5: Glycolysis inhibition combined with Akt or PI3K/mTOR inactivation displays high cytotoxicity to BCBL1 cells. BCBL1 cells were co-cultured for 24 or 48 hours with HMC, additioned or not with the indicated compounds. Annexin V stained cells were next analyzed by flow cytometry, as described in Methods section. A representative experiment is shown.

Supplementary Table S1: PEL cells were treated with increasing concentrations of NVPBEZ235, MK2206 and torin1. Then MTT assays were performed in triplicates, and the EC50 in normoxia (21%) and in hypoxia (1%) was calculated. Mean values and S.D. are indicated.

		BCBL1		HBL6	
**Drug**	**Molecular target**	**EC50 21% O2**	**EC50 1% O2**	**EC50 21% O2**	**EC50 1% O2**
**NVPBEZ235**	PI3K/mTOR	108.16±2.73nM	134.46±8.89nM	40.07±1.06nM	177.10±3.70nM
**MK2206**	Akt	1.3±0.34μM	1.25±0.42μM	2.5±0.65μM	2.56±0.55μM
**torin-1**	mTORC1/2	123.44±12.62nM	123.08±26.09nM		

Supplementary Table S2: See Supplementary Table S1 Optical density, measured with ImageJ software, of bands from, respectively: **(A),** Fig. 3C; **(B),** Fig. 4A left panel; **(C),** Fig. 4A right panel; **(D),** Fig. 2B SI; **(E),** Fig. 2C SI
