# Supplementary material for: Reversal of the glycolytic phenotype of primary effusion lymphoma cells by combined targeting of cellular metabolism and PI3K/Akt/ mTOR signaling
Source: Oncotarget. 2015 Nov 6;7(5):5521–37. doi: 10.18632/oncotarget.6315 (PMC4868703; doi:10.18632/oncotarget.6315)
Supplement: Supplementary file 2 [file oncotarget-07-5521-s002.doc]

|  | **HBL6** | | **BCBL1** | |
| --- | --- | --- | --- | --- |
|  | **Normoxia** | **Hypoxia** | **Normoxia** | **Hypoxia** |
| **mTOR S2448** | 57,6 | 42,4 | 62,0 | 38,0 |
| **mTOR** | 51,6 | 48,4 | 48,7 | 51,3 |
| **P70S6K T389** | 68,4 | 31,6 | 67,7 | 32,3 |
| **P70S6K** | 54,7 | 45,3 | 40,8 | 59,2 |
| **PDK1 S241** | 45,9 | 54,1 | 45,5 | 54,5 |
| **PDK1** | 37,2 | 62,8 | 38,0 | 62,0 |
| **Akt S473** | 51,1 | 48,9 | 53,7 | 46,4 |
| **Akt T308** | 46,7 | 53,3 | 42,1 | 57,9 |
| **Akt** | 48,3 | 51,7 | 49,4 | 50,6 |
| **Gsk3β S9** | 51,4 | 48,6 | 40,0 | 60,0 |
| **Gsk3β** | 47,1 | 52,9 | 43,3 | 56,7 |
| **Actin** | 52,0 | 48,0 | 49,8 | 50,2 |

Table 2A SI

|  | **Normoxia** | | | | **Hypoxia** | | | |
| --- | --- | --- | --- | --- | --- | --- | --- | --- |
|  | **DMSO** | **PF-04691502** | **NVPBEZ235** | **Akti 1/2** | **DMSO** | **PF-04691502** | **NVPBEZ235** | **Akti 1/2** |
| **P70S6K** | 10,4 | 9,0 | 9,7 | 7,4 | 7,8 | 21,9 | 12,0 | 21,0 |
| **P70S6K T389** | 28,4 | 11,7 | 7,2 | 1,4 | 19,7 | 6,5 | 18,3 | 6,9 |
| **Akt** | 20,9 | 17,3 | 9,6 | 10,8 | 6,5 | 9,7 | 12,7 | 12,5 |
| **Akt S473** | 19,1 | 6,8 | 17,6 | 4,9 | 16,1 | 11,9 | 14,6 | 9,1 |
| **Actin** | 10,1 | 8,2 | 9,3 | 10,6 | 10,8 | 6,2 | 12,4 | 12,6 |

Table 2B SI

|  | **Normoxia** | | | **Hypoxia** | | |
| --- | --- | --- | --- | --- | --- | --- |
|  | **DMSO** | **torin1** | **MK2206** | **DMSO** | **torin1** | **MK2206** |
| **P70S6K** | 18,9 | 16,4 | 16,9 | 13,2 | 15,6 | 19,0 |
| **P70S6K T389** | 30,8 | 2,7 | 16,6 | 23,8 | 2,0 | 24,1 |
| **Akt** | 13,0 | 15,1 | 18,9 | 17,7 | 18,2 | 17,1 |
| **Akt S473** | 17,3 | 20,7 | 18,3 | 20,8 | 7,6 | 15,0 |
| **Actin** | 14,5 | 16,0 | 16,4 | 18,3 | 18,7 | 16,1 |

Table 2C SI

|  | **Normoxia** | | | **Hypoxia** | | |  |
| --- | --- | --- | --- | --- | --- | --- | --- |
|  | **-** | **+** | **-** | **-** | **+** | **-** | **torin1, 75nM** |
|  | **-** | **-** | **+** | **-** | **-** | **+** | **rapamycin, 0.5µM** |
| **4EBP1 T37/46** | 26,1 | 7,0 | 20,4 | 23,1 | 16,4 | 27,5 |  |
| **4EBP1** | 12,5 | 17,1 | 19,8 | 21,2 | 4,3 | 19,1 |  |
| **Actin** | 13,7 | 11,1 | 21,8 | 22,3 | 17,9 | 11,5 |  |

Table 2D SI

|  | **4 hours** | | **24 hours** | |
| --- | --- | --- | --- | --- |
|  | **Normoxia** | **Hypoxia** | **Normoxia** | **Hypoxia** |
| **P70S6K T389** | 54,0 | 47,0 | 59,5 | 40,5 |
| **P70 S6K** | 56,5 | 43,5 | 42,4 | 57,6 |
| **AMPKα T172** | 33,5 | 66,5 | 21,2 | 78,8 |
| **AMPKα** | 51,4 | 48,6 | 48,1 | 51,9 |
| **REDD1** | 36,3 | 63,7 | 34,3 | 65,7 |
| **Actin** | 50,8 | 49,2 | 45,2 | 54,8 |

Table 2E SI
